# 7-Methylguanine Inhibits Colon Cancer Growth in Vivo

**DOI:** 10.32607/actanaturae.27422

**Published:** 2024

**Authors:** K. I. Kirsanov, T. I. Fetisov, E. E. Antoshina, T. G. Gor’kova, L. S. Trukhanova, S. I. Shram, I. Yu. Nagaev, Yu. A. Zolotarev, L. Abo Qoura, V. S. Pokrovsky, M. G. Yakubovskaya, V. K. Švedas, D. K. Nilov

**Affiliations:** Blokhin National Medical Research Center of Oncology, Institute of Carcinogenesis, Moscow, 115478 Russian Federation; RUDN University, Medical Institute, Moscow, 117198 Russian Federation; National Research Centre “Kurchatov Institute”, Moscow, 123182 Russian Federation; Lomonosov Moscow State University, Belozersky Institute of Physicochemical Biology, Moscow, 119991 Russian Federation; Lomonosov Moscow State University, Faculty of Bioengineering and Bioinformatics, Moscow, 119234 Russian Federation

**Keywords:** methylguanine, inhibitor, poly(ADP-ribose) polymerase 1, tRNA-guanine transglycosylase, colon cancer, BALB/c mice

## Abstract

7-Methylguanine (7-MG) is a natural inhibitor of poly(ADP-ribose) polymerase 1 and tRNA-guanine transglycosylase, the enzymatic activity of which is central for the proliferation of cancer cells. Recently, a number of preclinical tests have demonstrated the safety of 7-MG and a regimen of intragastric administration was established in mice. In the present work, the pharmacological activity of 7-MG was studied in BALB/c and BALB/c nude mice with transplanted tumors. It was found that 7-MG effectively penetrates tumor tissue and suppresses colon adenocarcinoma growth in the Akatol model, as well as in a xenograft model with human HCT116 cells.

## INTRODUCTION

7-Methylguanine (7-MG) is a nucleic acid metabolite that is found in small amounts in human blood and urine [1]. The study of 7-MG as a potential antitumor inhibitor began with virtual screening of natural nitrogenous bases and their derivatives against poly(ADP-ribose) polymerase 1 (PARP-1), a key DNA repair enzyme [2]. Modeling demonstrated complementarity between 7-MG and the PARP-1 active site, and further *in vitro *experiments confirmed the assumption about the competitive inhibition mechanism [3, 4, 5]. 7-MG also inhibits tRNA-guanine transglycosylase (TGT), an enzyme involved in the translation mechanism [6]. It was noted that knockout/knockdown of the TGT gene reduced the proliferation and migration of cancer cells [7].

The synthetic PARP-1 inhibitors olaparib, rucaparib, and niraparib are used in medicine as innovative anticancer drugs, but they come with serious side effects (in particular, myelodysplastic syndrome/acute myeloid leukemia) [8, 9]. At the same time, the natural inhibitor 7-MG demonstrated that it is safe in our toxicology study; a regimen of intragastric (i.g.) administration was established in mice – 50 mg/kg, 3 times per week [10]. The presence of several relevant targets (PARP-1, TGT) and the safety of 7-MG suggest prospects for further *in vivo *studies. This report describes for the first time the anticancer activity of 7-MG in colon adenocarcinoma models.

## EXPERIMENTAL

BALB/c mice (male, 4 weeks old) were obtained from the breeding of the Blokhin NMRCO. A sample of mouse adenocarcinoma Akatol [11] was obtained from the tumor collection of the Blokhin NMRCO. A suspension of tumor cells (0.5 ml, 0.1 g/ml) was subcutaneously injected into the suprascapular area. Treatment with the test compounds began on day 5 after inoculation. Mice were divided into groups of 9 animals each: control group I, water (i.g., 3 times per week); group II, cisplatin (2.5 mg/kg i.p., 2 times per week for 1 week); group III, 7-MG (50 mg/kg i.g., 3 times per week); and group IV, 7-MG + cisplatin. To prepare a 7-MG suspension (5 mg/ml), the compound was mixed with distilled water, vortexed, and left in an ultrasonic bath for 5 min at a temperature of 45°C. The resulting 7-MG suspension was administered by gavage. In combination treatment, 7-MG was administered 3 h prior to cisplatin. The volume of tumor formed was determined using the formula V = 1/2×length×width2. The analysis of the results was conducted after the average tumor volume in the control group reached 4 000 mm3.

A pharmacokinetic experiment was performed in male BALB/c mice on day 15 after Akatol inoculation. Mice were deprived of food for 18 h before the experiment. The animals were administered a single dose of 7-MG (50 mg/kg i.g.), and blood and tumor tissue samples were collected after 15 min (2 mice), 60 min (2 mice), and 180 min (3 mice). The samples were then frozen and subjected to lyophilization, mechanical grinding, and sequential extraction with solvents (aqueous 90% acetonitrile containing 2% trifluoroacetic acid, acetone, and aqueous 0.1% heptafluorobutyric acid). The quantitative analysis of 7-MG was performed by liquid chromatographymass spectrometry; an LCQ Advantage MAX mass spectrometer (Thermo Electron Co., USA) was used, equipped with a Surveyor Plus high-performance liquid chromatography system and an ESI ionization source. Deuterated 7-MG, obtained by solid state isotope exchange [12], was used as an internal standard.

Immunodeficient BALB/c nude mice (female, 6–7 weeks old) were obtained from the breeding of the Laboratory of Biochemical Fundamentals of Pharmacology and Tumor Models at the Blokhin NMRCO. A suspension of HCT116 human cancer cells (0.2 ml, 1.2 × 10^6^ cells/ml) was subcutaneously injected into both the right and left flanks of the mouse. Treatment with the test compounds began on day 10 after inoculation. Mice were divided into groups of 4 animals each: control group I, potassium phosphate buffer (i.p., 3 times per week); group II, cisplatin (1 mg/kg i.p., 3 times per week for 1 week); group III, 7-MG (50 mg/kg i.g., 3 times per week); and group IV, 7-MG + cisplatin. In combination treatment, 7-MG was administered 3 h prior to cisplatin. The tumor volume was determined using the formula V = π/6×length×width×depth.

All animal experiments were conducted in accordance with the requirements of the Local Blokhin NMRCO Committee for the Ethics of Animal Experimentation.

## RESULTS AND DISCUSSION

**Fig. 1:**
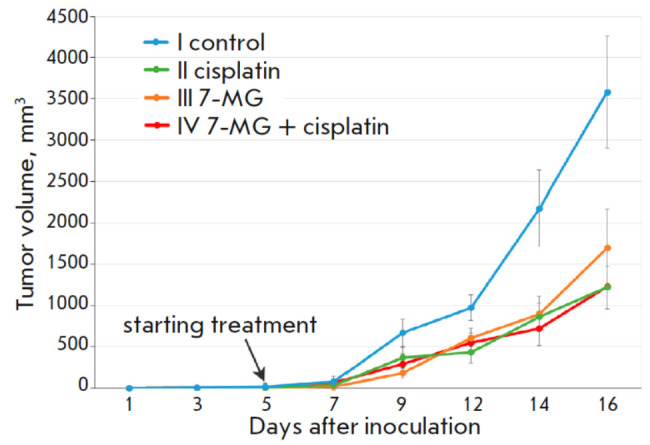
Dynamics of colon adenocarcinoma growth in the Akatol model

The biological activity of the 7-MG inhibitor upon i.g. administration was studied in the Akatol mouse model of colon cancer. The classic genotoxic agent, cisplatin, whose effectiveness had been previously demonstrated in the Akatol model, was used as a reference drug. On day 16 of the experiment, a significant inhibition of tumor growth by cisplatin (65.8%), 7-MG (52.5%), and their combination (65.5%) was observed (*Fig. 1*). The effects of the well-known chemotherapy drug cisplatin and the discovered PARP-1 inhibitor 7-MG were comparable, and the use of their combination did not appear to increase the antitumor activity.

**Fig. 2:**
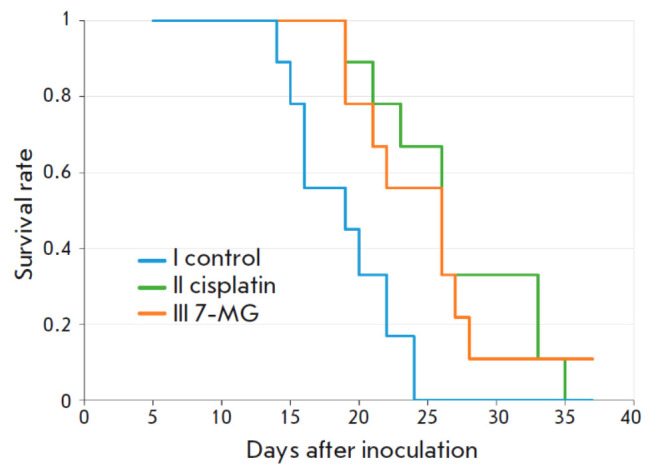
Survival of mice with an inoculated Akatol tumor (animals were excluded from the group when the tumor size reached 4 000 mm^3^)

According to guidelines for mouse handling, an animal can be excluded from the experimental group when a critical tumor size (4 000 mm3) has developed. The time required for the tumor to reach such a size can be considered as the animal’s survival after transplantation. *Figure 2* shows the survival curves, and that a significant increase in survival can be seen for the cisplatin and 7-MG groups.

To confirm the accumulation of 7-MG in the transplanted Akatol tumor tissue, a pharmacokinetic experiment was performed. The content of 7-MG in the tumor gradually increased following i.g. administration, and after 15, 60, and 180 min, it reached 218 ± 13, 460 ± 28, and 989 ± 59 ng/g, respectively. The ratio of 7-MG concentrations in the tumor and blood remained virtually unchanged and was on average 0.44, which, taking into account the low vascularization of the tissue, indicates effective tumor penetration of 7-MG.

**Fig. 3:**
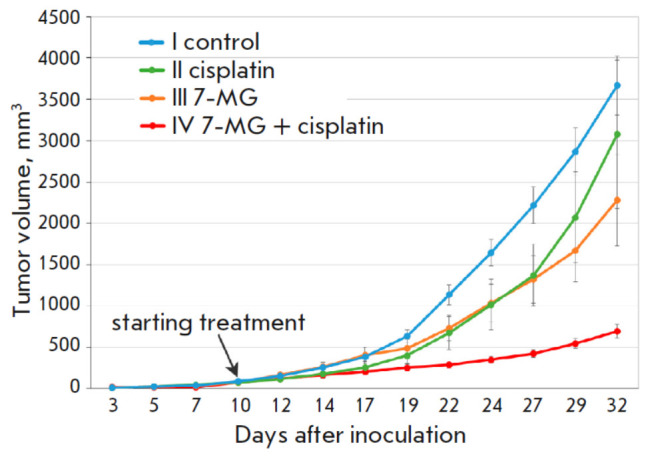
Dynamics of colon adenocarcinoma growth in a xenograft model

The antitumor activity of 7-MG was also tested in a xenograft model of colon cancer obtained by transplanting HCT116 human cancer cells into the mice. On day 32 of the experiment, cisplatin, 7-MG, and their combination inhibited tumor growth by 16.1, 37.8, and 80%, respectively (*Fig. 3*). Interestingly, the combination of 7-MG and cisplatin resulted in an additive effect that was not observed in the Akatol model. It is likely that HCT116 human cancer cells are more sensitive to a combined treatment with these two agents.

## CONCLUSIONS

An *in vivo *analysis of the antitumor activity of the natural compound 7-MG was conducted in the Akatol colon cancer model, as well as in a xenograft model with human HCT116 tumor cells. Inhibition of tumor growth with 7-MG treatment indicates the high effectiveness of the compound in an established regimen (50 mg/kg i.g., 3 times per week). Through a liquid chromatography-mass spectrometry analysis, high accumulation of 7-MG in tumor tissue was demonstrated. In the case of the xenograft model, the combined administration of 7-MG and the well-known chemotherapeutic drug cisplatin resulted in a significant increase in the antitumor effect (growth inhibition of 80%). The obtained data show promise for further studies of 7-MG as a new anticancer agent.

## Abbreviations

i.p.: intraperitoneal administration
i.g.: intragastric administration
7-MG: 7-methylguanine
PARP-1: poly(ADP-ribose) polymerase 1
TGT: tRNA-guanine transglycosylase
